# Dual-pathway mechanisms of plant-derived polysaccharides in ulcerative colitis: integrative roles of microbiota modulation, immune regulation, and barrier restoration

**DOI:** 10.3389/fphar.2026.1772007

**Published:** 2026-03-30

**Authors:** Yang Wenying

**Affiliations:** College of Bioscience and Biotechnology, Hunan Agricultural University, Changsha, China

**Keywords:** anti-inflammatory signalling, epithelial barrier repair, gut microbiota, plant-derived polysaccharides, short-chainfatty acids, ulcerative colitis

## Abstract

Chronic inflammation of the colon has been described as an inflammatory condition of the colon that is characterized by recurring flare-ups of the disease (ulcerative colitis). Ulcerative colitis is a complex inflammatory disorder of the colon that is determined by the combination of several mechanisms: abnormal bacterial flora, inappropriate immune response, and the breakdown of the protective lining of the colon. The last 10 years have seen the emergence of plant derived polysaccharides as new biological active compounds with the potential to intervene at the different levels of the disease. Plant derived polysaccharides can be divided into two separate regulatory modes; the first mode of action occurs when the polysaccharides work in collaboration with the microbiota to modify the structure of the microbiota and create a positive environment for the growth of the beneficial bacteria. The second mode of action occurs without the use of the microbiota and works by creating anti-inflammatory signals to regulate the body’s immune system and protect the integrity of the intestinal lining. The authors of this paper used a systematic method to identify 103 papers published on the subject of plant derived polysaccharides and their effects on the disease mechanism of ulcerative colitis. The results of the analysis of these papers indicated that the polysaccharides act to reduce the inflammatory process through the downregulation of the three major inflammatory signaling pathways of NF-κB, PI3K/AKT, and JAK/STAT. In addition, the polysaccharides were shown to restore the balance of macrophages and T-cells and increase the production of mucus producing cells and tighten the tight junctions between the intestinal epithelial cells. In recent studies it was found that the metabolic products of the microbes and the intracellular signaling processes are part of a coordinated regulatory network. Therefore, based on the results of the study, plant derived polysaccharides appear to be multi-target modulators of the disease process rather than just acting on one pathway of the disease process. Although the study indicates that there has been significant progress in understanding how the plant derived polysaccharides interact with the disease process, there are still many barriers to overcome before the plant derived polysaccharides can be used as a treatment for ulcerative colitis in humans. These barriers include; the structural heterogeneity of the polysaccharides, the lack of clinical trials evaluating the safety and efficacy of the polysaccharides as a treatment for ulcerative colitis, and the large variation in how individuals respond to changes in the composition of the gastrointestinal tract microbiota. Therefore, this study represents a comprehensive review of the current state of knowledge on the interaction of plant derived polysaccharides with the pathophysiology of ulcerative colitis, and highlights areas of future research necessary to develop polysaccharide based treatments for ulcerative colitis that are safe, effective, and can be translated into the clinic.

## Graphical Abstract

**Figure:**
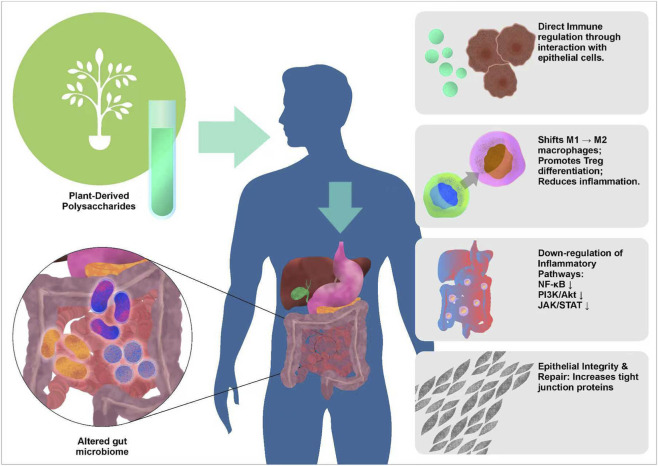
Created with Biorender(biorender.com).

## 1 Introduction

Ulcerative colitis (UC) is an ongoing inflammatory bowel disease (IBD) that impacts the colon. UC is defined as repeated periods of damage to the lining of the colon, in addition to unpredictable patterns of flare-up and remission, with continuous deterioration of the epithelial barrier. The incidence rate of ulcerative colitis has increased on a worldwide basis during the last 20 years; what was once primarily a western disease has become a global public health issue (Gros and Kaplan, 2023; Li W. et al., 2021; Segal et al., 2021; Kikut et al., 2022). The reasons for UC are a combination of a person’s genetic predisposition; how their immune system is disrupted; changes in their gut microbial populations; and environmental insult. The contributions of each of these components lead to a breakdown in homeostasis as it relates to the host and its microbiome, leading to a cascade of inflammation that damages the epithelial barrier further than initially damaged by the initial process of the disease and continues to further erode the tissue of the body (Irani et al., 2023).

UC results from a loss of regulation at several levels of control in the intestine; there is an abnormal activation of the body’s immune systems which results in increased production of pro-inflammatory cytokines and recruitment of effectors T-cells into the colonic mucosa with subsequent development of self-sustaining signaling pathways leading to continued epithelial damage (Tambovtseva et al., 2025; Li et al., 2024; Świrkosz et al., 2023; Zhang M. et al., 2023). When the epithelial layer is disrupted, a variety of substances (e.g., lumenal antigens and bacterial end products) are allowed to penetrate into the colonic mucosa and therefore intensify the local inflammatory response, thereby making the colon more susceptible to other external stimuli. Furthermore, changes in the gut microbiota composition, including loss of protective (commensal) bacteria and/or an increase in potentially pathogenic/opportunistic bacteria, also impede the metabolic and immunomodulatory effects afforded by commensal bacteria. Therefore, all these changes suggest a need for therapeutic interventions that affect disease activity at multiple levels with regard to UC (Nakase et al., 2022; Xiaohan and Zhanju, 2023; Koelink et al., 2024).

Polysaccharide-based compounds derived from plants have been of considerable interest in the treatment of UC as a result of their various biological properties, the great variety of structures that they can form, and their generally favorable safety profiles (Dilixiati et al., 2024; Li C. et al., 2021; Huo et al., 2025). Polysaccharides derived from plants are naturally occurring molecules made up of large (macro) molecular assemblies of complex combinations of monosaccharides. They can be found in many forms of natural substances such as plants, fungi, algae and other natural products. In contrast to most anti-inflammatory drugs that have been developed, which have primarily targeted a single pathway of inflammation, plant polysaccharides have demonstrated to modulate numerous biological pathways. As research continues to grow, it has become evident that polysaccharides exert their therapeutic effects via two modes of action; a microbiota dependent mode of action where polysaccharides alter the type of bacteria present and the metabolic by-products of those bacteria; and a microbiota independent mode of action where polysaccharides directly modulate the host’s immune response and reduce the host’s inflammatory response, while reducing oxidative stress and enhancing the integrity of the host’s mucous membrane lining. Therefore, because of the combination of these two mechanisms of action, polysaccharides have the ability to effect changes to both the host’s external microbial environment and its internal physiological systems, thus, creating a synergistic approach to improve colonic health and stability (Feng et al., 2022; Akanyibah et al., 2025; Li N. et al., 2023).

Research indicates that polysaccharides have the potential to alter gut microbiota composition and function through positive influences on the presence of beneficial, non-pathogenic microorganisms; inhibition of the proliferation of harmful bacteria; and restoration of the disrupted microbial equilibrium present in colitis. Moreover, the fermentation of polysaccharides by the gut microbiota results in the production of bioactive metabolites, including SCFAs and tryptophan metabolites, which can directly affect the maintenance of healthy epithelia, gene expression, and mucosal immunity. In addition to the microbiota-related mechanisms, polysaccharides may exert additional mechanisms independent of the microbiota, as they interact with signaling pathways relevant to the pathogenesis of UC, i.e., NF-κB, PI3K/AKT and JAK/STAT pathways. The utilization of these pathways allows polysaccharides to reduce inflammation, promote a balanced state between the innate and adaptive components of the immune system, enhance epithelial regeneration, and support the restoration of tight junction proteins necessary to maintain epithelial barrier function.

There is considerable evidence from multiple studies to suggest the efficacy of polysaccharides for treatment of UC; however, the scientific community still lacks detailed information regarding the structural-functional relationship of polysaccharides and the translational application of laboratory-based polysaccharide-based therapies to clinical settings (Ning et al., 2025). The DSS-induced rodent model is a most commonly employed model for studying the effects of inflammation on the gastrointestinal tract through dextran sulfate sodium (DSS) induced damage that has similarities to other forms of mucosal injury; however, it does not have all the complexity or variability associated with human diseases (Wang D. et al., 2024; Yang and Merlin, 2024; Xu et al., 2021). The variation in individual’s microbiota compositions also limits prediction of the outcome and standardization of the therapy protocol. However, the cumulative evidence from the 103 studies reviewed here represents substantial evidence that plant derived polysaccharides are a novel class of multi-target therapies to address the complex and multi-factorial pathology of Ulcerative Colitis (UC). This review is intended to synthesize the current mechanisms on how polysaccharides interact with microbiota-dependent and independent pathways, as well as integrate them into an overall comprehensive regulatory model; it will also discuss the existing challenges within the area and describe future areas of study which may enable accelerated translational advancement. By synthesizing all aspects of polysaccharide function, we anticipate providing a high level conceptual understanding that will be beneficial to the development of new generation polysaccharide based therapeutics for UC.

## 2 The microbiota-dependent mechanism of polysaccharide intervention

Colonic homeostasis is dependent on the dense interaction of the gastrointestinal microbiota. The microbiota supplies metabolic signals, immunologic signals, and structural signals to the colon for maintaining colonic homeostasis (Weis and Round, 2021; Choo et al., 2022; Lee et al., 2022; Li S. et al., 2023; Gustafsson and Johansson, 2022). However, in ulcerative colitis (UC), this relationship of the colonic microbiota has shifted away from what would be called a normal relationship to a state of dysbiosis. Dysbiosis involves an imbalance of the types of bacteria present in the colon with a decrease in beneficial commensals (such as lactobacilli) and an increase in pathogenic bacteria (Zakerska-Banaszak et al., 2021; Maldonado‐Arriaga et al., 2021; Molinero et al., 2022). Also, in UC there is impairment in the metabolic activity of the microbiota. Plant-derived polysaccharides may be able to restore a balanced relationship between the colonic microbiota and the colon and help to reduce the symptoms of UC by reducing the dysbiosis and helping to restore the metabolic function of the colonic microbiota. Polysaccharides appear to exert a large portion of their therapeutic effects by altering the structure of the colonic microbiota and thereby altering the output functions of the microbiota. These alterations occur via two interdependent mechanisms: structural modulation of the microbiota and regulation of the metabolites produced by the microbiota (Guo Y. et al., 2022; Pan et al., 2022; Yuan et al., 2023; Lu et al., 2023).

**FIGURE 1:**
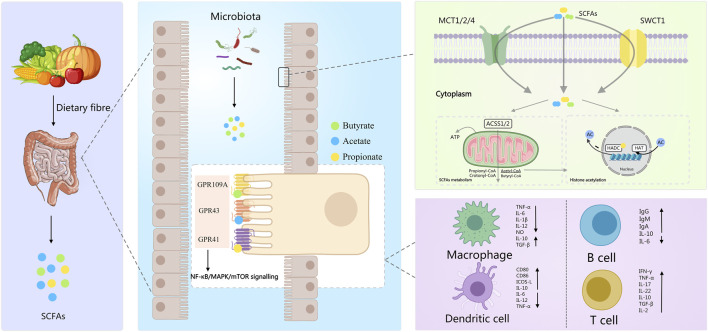
The molecular mechanism by which dietary fiber regulates immune cell function after being metabolized by the intestinal flora to produce short-chain fatty acids: Dietary fiber is decomposed by the intestinal flora to generate SCFAs. On the one hand, these ScFas activate signaling pathways such as NF-κB/MAPK/mTOR by binding to G protein-coupled receptors on the surface of intestinal epithelial cells. On the other hand, they enter cells via transport proteins and regulate gene expression through metabolic activation (such as ACSS1/2) or histone acetylation modification. Ultimately, it inhibits the release of pro-inflammatory factors from macrophages and dendritic cells, and regulates the immune responses of B cells and T cells, achieving precise regulation of the body’s immune function. Created with MedPeer (medpeer.cn).

Polysaccharides are a preferred substrate for many of the commensal bacteria found in the colon because they contain complex glycosidic linkages that are readily degraded by microbial glycosidases but poorly broken down by the mammalian digestive system. Therefore, when polysaccharides are introduced into a dysfunctional colon that is either inflamed or has developed a condition of dysbiosis, these molecules are able to selectively promote the growth of bacteria that are associated with the maintenance of mucosal barrier integrity and metabolic stability. A number of studies have demonstrated that the addition of polysaccharides to diets results in an increase in populations of beneficial bacteria, such as *Lactobacillus* spp., Bifidobacterium spp., Akkermansia spp., and families of butyrate-producing bacteria, all of which reside in the phylum Firmicutes. These beneficial bacteria contribute to the maintenance of colonic epithelial integrity by promoting mucus production, increasing the ability of colonocytes to produce energy, and regulating inflammation through the generation of various metabolites (Dilixiati et al., 2024; Ho Do et al., 2021; Cui L. et al., 2021). On the other hand, polysaccharides inhibit the growth of bacteria that cause inflammation of the epithelium or enhance the production of inflammatory mediators, including bacteria that generate endotoxin or proteolytically active metabolites. Through this selective modulation of the colonic microbiota, it is possible to re-establish a colonic microbiota that is more similar to the colonic microbiota seen in individuals without colonic disease. In addition, a large number of studies have shown that short-chain fatty acids (SCFAs) produced by the metabolism of dietary fiber through the intestinal flora can effectively maintain the structural integrity of the intestinal barrier through multiple pathways and play a key role in regulating the inflammatory response of the body (Xu et al., 2025). Figure 1 shows the generation, transport and absorption, intracellular metabolism and signal transduction pathways of SCFAs, as well as their regulatory processes on the functions of immune cells.

The polysaccharide-fermenting microorganisms’ effects are increased through the production of the SCFAs that occur as a result of their metabolism, especially acetate, propionate, and butyrate (Yang et al., 2022; Yu et al., 2023; Gu et al., 2023; Liu et al., 2023). SCFA’s play a significant role in intestinal homeostasis with the regulation of the intestinal epithelial barrier function by increasing tight junction proteins, reducing pro-inflammatory cytokine production (histone deacetylase inhibition), stimulating the mucosal immune system through G-protein coupled receptors (such as GPR43 & GPR109A) and functioning as the main energy source for colonocytes thereby facilitating epithelial cell turnover and regenerative processes (Shin et al., 2023).

Polysaccharide consumption typically leads to an alteration in the composition of the gut microbiome, leading to increased production of short-chain fatty acids (SCFAs) in response to this shift, and as such, is associated with clinical improvements, including decreased colonic inflammation, lower histological injury scores, and better stool quality. In addition to their ability to improve colonic environment through SCFA, they have been shown to promote the generation of regulatory T cells and to enhance the expression of anti-inflammatory cytokines to produce tolerance at the mucosa. The influence of SCFAs on the intestinal microenvironment represents a large portion of the polysaccharide-induced alterations in microbial metabolism; however, the polysaccharide-induced alterations in microbial metabolism may also alter the availability of secondary metabolites, including indole derivatives, which are produced during the catabolism of tryptophan. Indoles can interact with receptors involved in the modulation of mucosal immunity to enhance epithelial integrity and reduce inflammatory responses (Li F. et al., 2022).

The combination of the structural correction of the microbiota with the restoration of the metabolic functions of the microbiota is one of the main ways in which the beneficial aspects of the polysaccharide derived from plants affect the body’s physiological state, allowing it to achieve its therapeutic effects. The polysaccharides create a health-associated microbiota by creating a favorable environment for a health-associated microbiota to develop and enhancing the metabolic communication between the microbiota and the host, which is essential to maintain a stable epithelial barrier. Thus, the polysaccharides break the positive feedback loop of dysbiosis, inflammation, and mucosal damage present in ulcerative colitis. Hence, the improvement of the mechanisms dependent on the microbiota will allow for a favorable environment for the mechanisms independent of the microbiota to function optimally and this will be addressed in further sections. Therefore, the microbiota may be considered as the first site of therapeutic intervention using polysaccharides.

## 3 The microbiota-independent mechanism of polysaccharide action

Although the gut microbiome is a significant intermediate in the therapeutic effects of plant-based polysaccharides, they may also act directly on host tissues independent of microbial mediation. A second mechanistic route through which polysaccharides produce therapeutic effects is based upon how they modulate immune response, signaling networks within cells, oxidative stress management, and epithelial resilience; all of which are important in limiting the inflammatory cascade and tissue damage that characterize ulcerative colitis. The host-mediated effects of polysaccharides that do not depend upon the microbiome operate even when the structure of the microbiome is altered, thus illustrating the inherent bioactivity of polysaccharides on host physiology (Yuan et al., 2023; Huo et al., 2022; Bakky et al., 2025; Ying and Hao, 2023).

An essential mechanism of action of polysaccharides in this second pathway involves their potential to modify the behavior of immune cells (Zhao T. et al., 2025; Durairajan and Thangaraj, 2024). Ulcerative colitis is characterized by overactivation of both the innate and adaptive arms of immunity, with increased numbers of T-cells, macrophages, neutrophils, and dendritic cells infiltrating the inflamed colon (Kałużna et al., 2022). Plant polysaccharides are able to alter the direction of these responses using various immunological checkpoint points. For example, one of the most studied ways polysaccharides reorient immune responses is by promoting an appropriate ratio of activated macrophages. Typically, inflamed colonic tissue exhibits an excess of the pro-inflammatory macrophage type (M1) that secretes cytokines such as IL-1β, IL-6, and TNF-α. Polysaccharides induce the transition of this type of macrophage to the anti-inflammatory macrophage type (M2), while increasing the secretion of anti-inflammatory cytokines and promoting the healing process of damaged tissue (Zhang M. et al., 2023). The rebalancing of these types of macrophages helps restore tolerance of the immune system and decreases excessive inflammatory signaling.

Polysaccharides also influence T cell differentiation patterns. The most common pattern of T cell differentiation found in patients suffering from ulcerative colitis is an imbalance of Th17 cells and Tregs. Th17 cells contribute to the generation of cytokine storms and destruction of the epithelium, whereas Tregs maintain immune restraint and tolerance. Studies indicate that the exposure to polysaccharides stimulates the development of Tregs, decreases Th17 activity, and increases the production of IL-10, a crucial anti-inflammatory cytokine. Collectively, these immune-balancing effects decrease inflammation more generally and prevent the chronic activation cycles that lead to the progression of colitis (Huang C. et al., 2022; Zhang Y. et al., 2023; Hu et al., 2025).

A second primary mechanism of action of polysaccharides is to regulate the signaling pathways that determine the expression of genes involved in inflammation, oxidative stress, cell death and turnover of epithelial cells, and repair of the mucosa. There are three signaling pathways that are particularly relevant to the study of polysaccharide effects because of their key roles in regulating inflammatory programs, oxidative stress responses, epithelial cell turnover, and mucosal repair: the NF-κB, PI3K/Akt, and JAK/STAT signaling pathways (Yuan et al., 2023; Zhang S. et al., 2023; Zeng et al., 2024; Shan et al., 2024).

NF-κB is a transcription factor that is responsible for the regulation of the expression of cytokines, chemokines, adhesion molecules, and inflammatory mediators. Activation of NF-κB is often elevated in the context of colitis, leading to continuous inflammation. Several studies demonstrate that polysaccharides can inhibit the movement of NF-κB into the nucleus from the cytoplasm by either stabilizing its inhibitor, IκBα, or by inhibiting the upstream kinases that promote the propagation of inflammatory signals (Huo et al., 2022; Zeng et al., 2024; Wang Y. et al., 2024). These actions result in decreased transcription of inflammatory cytokines and lessened inflammatory burden in the mucosa. Such inhibition of NF-κB signaling acts independently of microbial modification, indicating that polysaccharides can modulate inflammatory signaling circuits within the cell directly.

The PI3K/Akt pathway is another signaling target of polysaccharides. The PI3K/Akt pathway controls cell survival, proliferation, and barrier function. Abnormal activation of the PI3K/Akt pathway in ulcerative colitis leads to dysregulated epithelial cell turnover and sensitization to inflammation. Polysaccharides are capable of normalizing the signaling of the PI3K/Akt pathway by decreasing the phosphorylation of Akt and normalizing epithelial cell responses. By doing so, polysaccharides help to preserve the integrity of the epithelial barrier and limit the loss of barrier function observed in colitis (Zhang et al., 2024; Ma et al., 2023).

Similar to the PI3K/Akt pathway, the JAK/STAT pathway, particularly STAT1 and STAT3 signaling, has a significant role in modulating cytokine responses and maintaining the balance between inflammation and repair (Huang B. et al., 2022). Overactivated STAT leads to amplified inflammatory cascades and prevents mucosal healing. Polysaccharides can decrease STAT phosphorylation, therefore dampening the expression of inflammatory mediators and supporting the regeneration of epithelial cells (Sarapultsev et al., 2023). These effects demonstrate the immunomodulatory capacity of polysaccharides and illustrate the capability of polysaccharides to directly affect inflammatory networks as shown in Table 1.

**TABLE 1:** Key microbiota-independent targets influenced by plant-derived polysaccharides.

Polysaccharide Source (Example)	Primary Pathway Modulated	Cellular/Immunological Outcome	Overall Effect on Ulcerative Colitis
Root-, berry-, or seed-derived polysaccharides (Narwal et al., 2024)	NF-κB inhibition	Reduced transcription of IL-6, TNF-α, IL-1β; lowered inflammatory gene expression	Decreased mucosal inflammation and reduced flare intensity
Herbal or fruit polysaccharides (Arora et al., 2024)	Promotion of Treg differentiation; reduced Th17 activity	Restored immune tolerance; increased IL-10; balanced adaptive immunity	Suppressed chronic inflammatory cycles; improved mucosal healing
Starch-, tuber-, or grain-derived polysaccharides (Liu, 2024)	PI3K/Akt regulatory effects	Stabilized epithelial turnover; enhanced survival, and moderated oxidative stress	Strengthened barrier integrity and reduced epithelial erosion
Fungal or algae-based polysaccharides (Asowata‐Ayodele et al., 2023)	JAK/STAT suppression	Reduced STAT1/STAT3 phosphorylation; decreased inflammatory cytokine signalling	Attenuated inflammation and improved epithelial recovery

As well as modulating the immune response and down-regulating inflammatory signals, the anti-oxidant activity of polysaccharide is an additional mechanism of action that is separate of the microbiota (Zhao L. et al., 2025; Shen et al., 2022; You et al., 2024). Oxidative stress has been determined to be one of the factors causing mucosal injury and malfunction in patients with ulcerative colitis. Oxidative stress occurs due to elevated amounts of ROS which disrupt tight junction proteins, reduce epithelial generation and increase cytokine signaling (Afzal et al., 2023). Each of the polysaccharides reviewed have shown significant anti-oxidant properties through the enhanced production of the body’s endogenously produced anti-oxidant enzymes including SOD, GPx and Catalase. The enhanced anti-oxidant status of the mucosa lessens oxidative stress and protects the epithelial cells from injury while providing a better environment for the healing of damaged tissues.

Polysaccharides help to create a stronger, more intact mucosa by increasing the overall integrity of the epithelium. Independent of interactions with the microbiome, polysaccharides are known to increase proliferation of epithelial cells; decrease the rate of apoptosis (or cell death); and produce more mucus (Huo et al., 2022; Sauruk da Silva et al., 2021; Yan et al., 2024). The increased mucus produced will create an additional layer of protection for the epithelial layer, providing a barrier against other irritants found in the lumen. Additionally, polysaccharides can increase the expression of the tight junction proteins (occludin, claudin-1 and ZO-1) that keep the epithelial layer physically intact. Therefore, like immune modulation and anti-inflammatory properties, these polysaccharide-induced changes result in restoration of the structural integrity of the colon (Gieryńska et al., 2022; Liu et al., 2021; Yu et al., 2025).

Through both microbiota-dependent and independent mechanisms of action, the multitude of effects that polysaccharide molecules from plants have on ulcerative colitis create a solid foundation upon which the polysaccharides can influence this condition. The polysaccharides can modulate many different pathways by which they exert an anti-inflammatory effect and promote mucosal healing. In addition to their previous effects on the metabolism and ecology of the gut microbiota, these host-directed mechanisms offer a basis for developing a multifaceted therapeutic strategy using a two pronged approach that will be explored further in the next chapter.

## 4 Intestinal barrier repair

The intestinal epithelium is the body’s initial line of protection from its external environment (Jutfelt and Sundh, 2024), the chronic inflammation seen in ulcerative colitis has destroyed the balance of the barrier function of the epithelial lining and allowed for the presence of microbial translocation of toxic substances and antigens through this lining. This results in an ongoing cycle of inflammation which damages tissues and inhibits the ability to heal. Plant-derived polysaccharides aid in repairing the damaged mucous membrane by directly interacting at the molecular, structural and biochemical level with the epithelial interface. Plant-derived polysaccharides can be used to induce regeneration of epithelial cells; increase mucus synthesis; increase the strength of the tight junctions between epithelial cells; and enhance the production of protective enzymes (Huo et al., 2022; Yan et al., 2024; Guo S. et al., 2022; Shen et al., 2023).

The four linked barriers which constitute an intact intestinal barrier (Di Tommaso et al., 2021; Cui M. et al., 2021) are mechanical, chemical, immune and microbial. Mechanical barrier functions through the connections between epithelial cells and their tight junctions; the chemical barrier operates through mucin secretion and antimicrobial peptide secretion; the immune barrier functions through secretory IgA and innate immunity; and the microbial barrier exists through the presence of commensal bacteria on the mucosa. When someone suffers from active colitis, each of the above-mentioned barriers will be compromised in such a way as to allow for an increase in “leakiness” or permeability and an atrophy of the epithelial cells along with loss of tolerance of the immune system (Boles et al., 2024; Yu S. et al., 2022; Sun et al., 2024). The pathological pattern of decreased function of the barrier may be corrected by initiating repair cascades that help the epithelial cells adhere better and/or produce protective materials.

Polysaccharides can also stimulate the proliferation of intestinal stem cells and induce them to develop into either absorptive or goblet cells; thus stimulating the regeneration of the epithelium. This stimulates the regeneration of the epithelium to heights of villi and depths of crypts greater than those of injured epithelia. In addition, polysaccharides stimulate the production of growth factors such as EGF and TGF-β that are involved in the repair of damaged epithelial cells and inhibit excessive epithelial cell proliferation, which is potentially harmful and inflammatory. The increased goblet cell differentiation results in an increase in mucin secretion (which increases the thickness of the mucus layer) which physically impedes the irritating effects of luminal irritants from contacting the epithelial surface (He et al., 2021).

Polysaccharides also have been proven to repair tight junctions destroyed by inflammatory cytokines (such as TNF-alpha & IFN-gamma) (Liu et al., 2021; Li Z. et al., 2022). Which damaged the proteins forming the tight junctions including occludin, claudins, and ZO-1; these proteins create the molecular seals controlling the permeability of the paracellular space. The decrease in the protein expression of these tight junction proteins created an increase in intercellular spaces. The polysaccharide molecules blocked the signaling pathways responsible for the promotion of inflammation and enhanced the transcription of tight junction proteins restoring tight junctions and reducing the permeability of the mucosal surface and preventing further antigens from entering the body through the mucosa. Some polysaccharides also increased the phosphorylation of myosin light-chain kinase in a controlled manner to enhance the stability of the cytoskeletal structure of epithelial cells and strengthen the epithelial cell junctions without inducing contraction or stress.

The protective mechanism against barrier injury also involves decreasing oxidative stress. The mucosa in the region of inflammation produces high levels of reactive oxygen and nitrogen species, which destroy the membrane lipids and damage the tight junctions between the epithelial cells. The polysaccharides have antioxidant characteristics that enable them to eliminate free radicals and initiate the activation of the body’s own protective mechanisms, including superoxide dismutase (SOD), catalase, and glutathione peroxidase (GPx). The dual action of polysaccharides enables them to limit oxidative damage and the resultant lipid peroxadation and preserve the fluidity of the cell membranes. Generally, the antioxidant action of polysaccharides results from their high content of hydroxylic groups, which enable the transfer of hydrogen atoms to neutralize the free radicals and subsequently halt the progression of oxidative chain reactions within the epithelial cells.

As well as providing structural and oxidative protection, polysaccharides may affect the chemical barrier by stimulating the production of mucins and antimicrobial peptides. Mucin-2 (MUC2) is the main gel forming glycoprotein involved in producing the mucus layer overlying the intestinal epithelium. The loss of MUC2 and goblet cells in the area of inflammation exposes the epithelial cells to luminal substances. The administration of polysaccharides has resulted in increased mRNA expression for MUC2, an increase in the number of goblet cells, and thickening of the mucus layer. In addition, polysaccharides may enhance the expression of defensins and lysozymes, two innate antimicrobial peptides that inhibit luminal bacteria and contribute to maintaining microbial equilibrium on the surface of the epithelial cells.

Improvement of the barrier integrity of the gut mucosa caused by polysaccharides may also be supported by the interaction between the immune system and epithelial layer (Yao et al., 2024). Secretory IgA produced in the lamina propria will support mucosal immunity without provoking an overt inflammatory response. Polysaccharides will help facilitate the exocytosis of secretory IgA to the lumen and help maintain the viability of plasma cells in Peyer’s patches to provide a second line of defense against infection for the mucosa as a whole; once secretory IgA is present it will neutralize the toxic effect of microorganisms prior to their interaction with an epithelial cell receptor thereby preventing secondary infections and subsequent inflammatory responses (Bamias et al., 2023; Strugnell, 2022).

**FIGURE 2:**
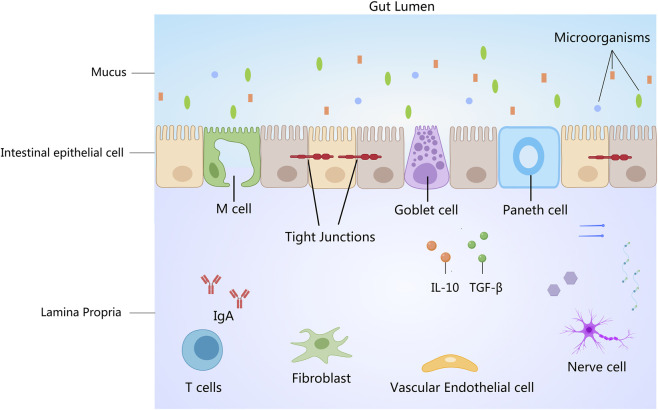
The intestinal barrier under human health conditions. Created with MedPeer (medpeer.cn).

In addition, TJs, a key component of the intestinal mucosal epithelial boundary, is closely related to barrier integrity and associated inflammation. Certain phytochemicals can enhance membrane permeability and integrity by restoring TJs levels (Panwar et al., 2021). Figure 2 shows the intestinal barrier under human health conditions.

The interrelatedness of the individual components of restoration is illustrated through both the clinical and laboratory data that have been generated. The efficacy of polysaccharides as a therapeutic agent has been demonstrated in multiple studies to decrease measures of colon permeability, normalize serum levels of endotoxins, and reverse the tissue changes found in ulcerative colitis. Histological findings revealed significantly less damage to epithelial cells, reduced numbers of infiltrating leukocytes and increased continuity of mucous throughout the crypts. Mechanistic improvements were made possible due to the enhanced structural integrity of the epithelial layer which restricted the translocation of luminal bacteria into the tissues and created an environment where beneficial anaerobic bacteria could reside (i.e., preserved the oxygen gradient) creating a basis for the long term stabilization of the intestinal microflora (Huo et al., 2022; Yan et al., 2024; Cui M. et al., 2021; Peng et al., 2024). Therefore, this illustrates how the structural repair of the barrier function is interdependent upon the ecologic balance of the gut flora.

Another important component of the recovery of barrier function involves the signal transduction pathways responsible for the regulation of apoptosis and autophagy in the epithelial cells. The apoptosis process could be involved in a thinning of the epithelial layer. In addition to maintaining cellular recycling and nutrient handling, autophagy is an essential part of the maintenance of cellular health. Autophagy is activated by polysaccharides, and activated through AMPK (adenosine monophosphate-activated protein kinase) and inhibition of mTOR (mechanistic target of rapamycin); with the concomitant inhibition of apoptosis due to inflammation through reduced caspase-3 and Bax protein expression (Wu et al., 2025; Wan et al., 2022; Yu L. et al., 2022). Cellular turnover can then be regulated to provide sufficient numbers of new cells to replace dying cells without too much cell loss disrupting barrier integrity. Also, controlled autophagy will help remove damaged organelles and misfolded proteins to increase cellular efficiency during the recovery process.

These microbiota-independent and epithelial-related mechanisms indicate that polysaccharides obtained from plants are not just passive barriers; they can also act as a biological modulator to coordinate the activation of simultaneously functioning regeneration, antioxidant and immunomodulatory systems in order to maintain mucosa homeostasis. Restoration of the mucosal layer barrier prevents the repeated episodes of inflammation and creates the ability to regulate the relationship between microorganisms and the host’s immune system over time. The next portion of this paper is going to describe how the two types of pathways (microbiota dependent and independent) create an integrated system that represents the basis of the broad therapeutic use of polysaccharide containing extracts derived from plants in the treatment of ulcerative colitis.

## 5 Cross-talk between dual pathways: an integrative mechanistic framework

While polysaccharides from plants have demonstrated therapeutic properties for ulcerative colitis through both microbiota-dependent and microbiota-independent mechanisms, a comprehensive understanding of how they work together in concert with one another is necessary to understand how they produce therapeutic results.

Both of these mechanisms interact with one another; biochemical signals, cellular processes, and interactions at the ecosystem level provide the feedback loops between them.

The unifying regulatory network created by the mechanisms described above provides a framework for the regulation of the host’s physiological responses to microbial products, the host’s immune response to changes in the microbial community, and the maintenance of the homeostasis of the microbial community.

Therefore, the interdependence of these various components of this network is important to understanding the wide therapeutic range of polysaccharide compounds in the treatment of ulcerative colitis.

An important aspect of the interrelationship of these mechanisms of action is the role of short-chain fatty acids (SCFAs), produced as a result of microbial fermentation of polysaccharides. As mentioned previously, SCFAs (butyrate and propionate) have direct beneficial effects on inflammation and maintaining the integrity of the epithelial layer. However, the significance of SCFAs extend beyond their individual roles in being beneficial. SCFAs serve as significant intermediary molecules between the metabolic activities of microbes and the host. SCFAs inhibit histone deacetylase enzymes, thereby inhibiting the transcription of genes involved in inflammation within epithelial and immune cells. Furthermore, SCFAs activate G-coupled protein receptors (GPCRs) on colonocytes and immune cells, and activation of these receptors results in mucosal repair and immunologic tolerance. Thus, SCFAs act as biochemical mediators of host-directed therapeutic signals, converting the metabolic activities of microbes into signals that direct the host towards therapeutic outcomes (Che et al., 2023; Li J. et al., 2022; Zhang et al., 2025; Liu et al., 2023).

There is another important connection point between these two processes in the form of a relationship involving “immune-microbial feedback loops.” The gut immune system controls microbial populations by releasing antimicrobial peptides, immunoglobulins, and cytokines which determine the type of microbes that will be able to survive in the gut. Certain types of polysaccharides are known to promote regulatory immune functions—primarily via stimulation of T-regulatory (Treg) function—and can thus limit the amount of inflammation present in the body. By limiting inflammation, it creates an environment that is conducive to the growth of various beneficial bacteria, as they then create products (metabolites) that help to regulate immune homeostasis. In addition to creating a favorable environment for the growth of beneficial bacteria, the stimulation of secretory IgA caused by polysaccharides also encourages colonization of beneficial bacteria, while inhibiting the ability of pathogenic bacteria to adhere to the epithelial surface of the intestines. Together these mechanisms illustrate the cooperative nature of the two distinct pathways (Yang et al., 2025; Pavithra et al., 2026; Sun et al., 2025).

Moreover, the integrity of the epithelial layer represents yet another site of convergence. A functioning mucosa barrier protects host tissue and is required to create the environment rich in oxygen gradients needed for the growth and maintenance of commensal anaerobic bacteria. The barrier functions through increases in tight junction expression; by enhancing epithelial cell turnover; and through increasing mucus secretion, which are mediated by polysaccharides. With the restoration of the barrier, the rates of both microbial translocation and inflammatory signaling are reduced. These reductions in inflammation modify the luminal environment, and promote the return to dominance of commensals capable of producing short chain fatty acids (SCFAs) and degrading mucins. These commensals produce metabolites that further enhance barrier function and interact with host pathways (AMPK, GPR109A and TLRs). Therefore, polysaccharide mediated repair of the epithelial barrier establishes the conditions that are conducive to the recovery of microbial communities, and these recovered microbial communities enhance the protective capabilities of the epithelial layer.

However, SCFAs represent only one type of metabolite that mediate communication between the microbial community and the host. Another important group of metabolites that participate in this communication include the tryptophan-derived indole compounds. These compounds interact with the aryl hydrocarbon receptor (AhR), a transcription factor that regulates the process of epithelial cell renewal, IL-22 secretion, and the defense of the epithelial layer from pathogens. When polysaccharides induce a shift in microbial metabolism to produce greater amounts of indoles, AhR signaling is activated and this produces elevated levels of antimicrobial peptide synthesis and enhanced resistance of the epithelial layer to injury. Thus, the synergy between the two pathways described herein is not limited to SCFA production but includes all types of aromatic metabolic pathways that have a significant impact on mucosal immunity.

Lastly, the inflammatory signaling pathways, including those involving NF-κB, JAK/STAT and PI3K/Akt, provide a third set of convergence points for the previously mentioned mechanisms. Directly, microbial metabolites can modulate these pathways, and indirectly, polysaccharides can modulate these pathways via receptor-mediated signaling. In a well-coordinated system, beneficial microbial metabolites will decrease NF-κB activation, whereas polysaccharides will inhibit the upstream kinases responsible for activating NF-κB. In addition, SCFAs will inhibit the hyper-phosphorylation of STAT3 and STAT1, whereas polysaccharides will independently downregulate STAT phosphorylation. The overlapping of these mechanisms produces redundant protective layers that ensure the optimal functioning of inflammatory pathways. Typically, redundancy in biological systems is advantageous because it allows for the stability of the system regardless of the fluctuation in either the environmental or pathological state. To illustrate how these interactions converge, the following table outlines the primary nodes of interaction among the microbial and host-directed processes. This table illustrates how polysaccharides interact with multiple systems simultaneously, resulting in cumulative therapeutic effects as shown in Table 2.

**TABLE 2:** Integrative interaction nodes between microbiota-dependent and microbiota-independent pathways.

Interaction Node	Microbiota-Dependent Driver	Host Pathway Influenced	Combined Biological Effect
SCFA production (butyrate, propionate) (Xu et al., 2022)	Fermentation by beneficial anaerobes	GPCR signalling, HDAC inhibition	Reduced inflammation, enhanced barrier integrity
Indole derivatives from tryptophan metabolism (Liu et al., 2023)	Microbial aromatic metabolism	AhR activation	Increased IL-22, antimicrobial peptide production, epithelial regeneration
Restored goblet-cell function (Singh et al., 2022)	Microbial balance promoting mucin synthesis	MUC2 transcription and mucus assembly	Strengthened chemical barrier and reduced luminal irritation
Tight-junction reinforcement (Liu et al., 2021)	Microbial suppression of oxidative stress	Upregulation of occludin, claudins, ZO-1	Reduced permeability and lower inflammatory translocation
Balanced immune cell subsets (Yang et al., 2025)	SCFA-driven Treg expansion	Suppression of Th17 and inflammatory cytokines	Sustained mucosal tolerance and decreased relapse risk
Epithelial energy metabolism (Lan et al., 2023)	Butyrate as colonocyte fuel	AMPK activation and reduced apoptosis	Improved epithelial survival and tissue recovery

Polysaccharide’s dual pathway modulatory effects show how the polysaccharides regulate the multiple physiological systems in harmony. Polysaccharides can affect many biological pathways such as the ecological status of microbes, intestinal (epithelial) function, and immune response and through their collective effect on multiple biological systems can offer a wide range of therapeutic advantages over a single pathway approach. These interactions are probably responsible for the fact that polysaccharides have demonstrated anti-inflammatory properties, restored/repair the mucosa, normalized the microbiome, etc., in many experimental models.

Emerging data increasingly indicate that the context influences the dual pathway interaction. For instance, if there is a high level of (severe) disruption to the natural balance of the gut microbiota (i.e., dysbiosis), the host’s pathways could possibly compensate for the lack of available microbial metabolites until the microbiota regains a more balanced or “natural” state. However, when the level of dysbiosis is lower (mild), then the metabolite products from the microbiota would likely dominate the repair of damaged tissue and the host’s pathways would provide supporting auxiliary functions. The flexible mechanistic structure of the dual pathway synergy affords the assurance that polysaccharides have the potential to be effective therapeutics regardless of the level of disease severity.

In conclusion, the dual pathway synergy of plant derived polysaccharides has provided a theoretical framework which has integrated the many separate microbial, immunologic and epithelial functions into a unified therapeutic model that stabilizes the colonic physiologic environment while preventing recurrent inflammatory events and maintains the integrity of all components of the mucosal defense system. The next section discusses the current obstacles to developing consistent and clinically relevant therapies based upon these principles and identifies critical knowledge gaps that must be filled before further clinical applications are feasible.

## 6 Limitations and research gaps

Polysaccharide-induced modification of the dual pathways illustrates how polysaccharides coordinate interconnected physiological systems. As such, polysaccharides influence multiple biological pathways which include the ecological status of microbes, the intestinal (epithelial) function and the immune response. Therefore, the resultant synergistic effect on many biological systems leads to significant therapeutic advantages over the use of a single pathway. There is increasing evidence to suggest that the dual pathway interaction is dependent on the context. For example, when the normal balance of gut microbiota is significantly disrupted (dysbiosis), it is possible that the host’s pathways will compensate for the lack of available microbial metabolites until the microbiota returns to a more stable or ‘normal’ state. Conversely, when the degree of dysbiosis is less severe (mild), the metabolic products from the microbiota may play the major role in repairing damaged tissue, while the host’s pathways may provide supportive assistance. This flexibility of the structural mechanism of the dual pathway synergy ensures that polysaccharides will have the capability to effectively treat diseases at every level of severity.

Therefore, in summary, the dual pathway synergy between plant-derived polysaccharides has provided a theoretical basis to combine the functions of microbial, immunological and epithelial components into a coherent therapeutic model that both stabilizes the physiological environment of the colon, as well as prevents recurrent inflammatory episodes and maintains the structural integrity of each component of the mucosal defense system. The following section addresses the current challenges to translating these concepts into consistent and clinically applicable therapies and identifies key areas of knowledge that need to be filled to enable clinical translation.

## 7 Future directions for translational advancement

To advance the translation of polysaccharides from laboratory research to practical therapeutic products for ulcerative colitis (UC), a multi-dimensional integrated research strategy must be established to bridge the gap between basic research and clinical application.

The core lies in clarifying the structure-activity relationship (SAR) of polysaccharides. Utilizing sophisticated techniques such as nuclear magnetic resonance (NMR) spectroscopy and mass spectrometry (MS), combined with molecular docking and machine learning models, we aim to decipher the correlation between structural characteristics (e.g., monosaccharide composition and molecular weight) and biological activities, thereby identifying key structural motifs that mediate anti-inflammatory effects. Concurrently, it is essential to optimize colon-targeted delivery systems: novel formulation technologies can prevent polysaccharide degradation in the upper gastrointestinal tract, or composite delivery systems integrating polysaccharides with probiotics or anti-inflammatory agents can be constructed to achieve precise targeting and synergistic therapy.

The integrated application of multi-omics technologies is critical for mechanistic exploration. Through metagenomics, metabolomics, and other omics approaches, we can identify biomarkers of treatment response and anti-inflammatory metabolic pathways, as well as establish therapeutic efficacy prediction models. Personalized medicine should be based on individual gut microbiota profiles, with machine learning algorithms customizing polysaccharide types and dosages to avoid a “one-size-fits-all” approach.

At the clinical translation stage, large-scale, multi-center randomized controlled trials (RCTs) are required. Study designs should incorporate stratification by disease severity and microbial characteristics, with evaluations focusing on core endpoints such as mucosal healing and relapse prevention, alongside long-term safety. Simultaneously, universal guidelines and pharmacopoeial standards for polysaccharide preparation and purity testing must be established, and scalable, low-cost extraction processes developed.

Furthermore, the combined application of polysaccharides with conventional therapies should be explored to verify their adjuvant value in reducing toxicity and enhancing efficacy. In-depth research into the regulatory mechanisms of polysaccharides on the gut-brain axis and gut hormones is needed to identify novel therapeutic targets. *In vitro* models such as intestinal organoids can be utilized to dissect the interactions between polysaccharides and the epithelium-immune-microbiota triad.

Through interdisciplinary collaboration, polysaccharides will ultimately be transformed into mechanism-defined, safe, and effective personalized targeted therapeutics for UC.

## 8 Conclusion

While many pharmaceutical compounds have been researched as treatments for Ulcerative Colitis (UC) and numerous studies have demonstrated the therapeutic activity of these drugs, no drug compound has been shown to be as therapeutically active as Plant-Derived Polysaccharides (PDPs). The reasons for this are twofold: PDPs interact with both the luminal environment of the gastrointestinal tract (via effects such as SCFA production, indole derivative production, tight junction regulation, etc.), and they also interact directly with the host (through enhanced epithelial barrier function, decreased oxidative stress, etc.). This means PDPs can assist in restoring homeostasis to an individual’s luminal microenvironment, while also contributing to the repair of damaged host tissues, which is necessary to reduce the ongoing and chronic nature of UC.

Another advantage of PDPs is their ability to generate a synergistic interaction between luminal and host-directed mechanisms of action. For example, in the presence of PDPs, the elevated levels of beneficial metabolites such as SCFAs, indoles, and others produced by a healthy population of bacteria within the colon contribute to the repair of damaged epithelial cells, modulate the immune response, and restore normal function to the intestinal epithelium. As noted earlier, the synergy mentioned above contributes to a multi-factorial therapeutic outcome that promotes the resolution of inflammation and the promotion of mucosal healing in individuals suffering from active UC.

Beyond the benefits mentioned above, another advantage of PDPs is that they are relatively inexpensive, and readily accessible. However, before PDPs can be utilized effectively in humans several issues must be resolved. These issues include the structural heterogeneity of each type of PDP, the variability of methodologies employed to assess the effectiveness of PDPs, and the lack of multi-omics approaches and well-designed clinical trials assessing the effectiveness of PDPs in humans. Therefore, in order to develop PDPs for the treatment of active UC, it will be necessary to employ advanced methodologies to determine the structure of PDPs, improve delivery systems for PDPs, develop personalized therapeutic algorithms based upon an individual’s microbiota profile, and conduct well-designed, multi-center, and randomized clinical trials to evaluate the safety and efficacy of PDPs when delivered in a controlled fashion.

Ultimately, PDPs may represent a new class of multi-functional therapeutic agents capable of addressing multiple aspects of the pathophysiology of active UC. Therefore, if PDPs undergo additional scientific investigation and clinical evaluation using standardized methodologies, they may provide a new generation of safe, effective, and personalized therapeutic agents that can be used in conjunction with current UC therapies.
